# Public health round-up

**DOI:** 10.2471/BLT.19.010219

**Published:** 2019-02-01

**Indonesia tsunami response**

National and provincial authorities continue to assist people affected by the tsunami which hit Indonesia on 22 December 2018, breaking along the Sunda Strait and devastating Banten and Lampung provinces. According to Indonesia’s National Agency for Disaster Countermeasures, 437 people were killed, and 14 059 injured. This photo shows residents searching through the rubble to salvage possessions from their home that was damaged during the tsunami in Tanjung Jaya Village, Panimbang District, Pandeglang, Banten, in the western part of Java.

**Figure:**
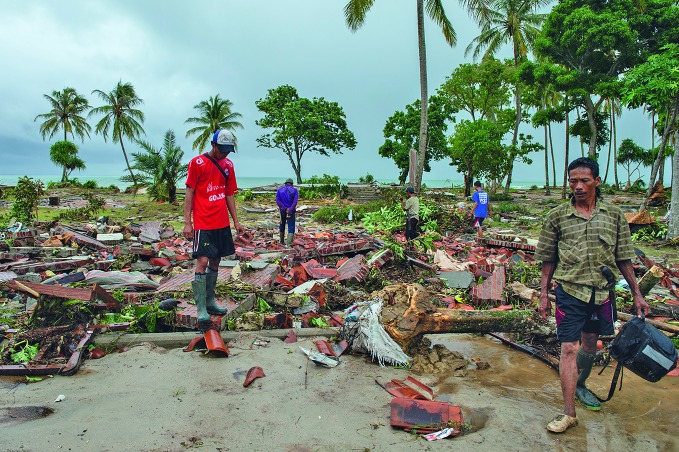
UNICEF/Arimacs Wilander

## WHO forms gene editing advisory group

The World Health Organization (WHO) is setting up an expert advisory group to develop global standards for governance and oversight of human gene editing. 

The move follows the recent application of the new CRISPR-Cas9 technology to edit the human genome. 

The technique uses an enzyme to edit gene sequences. The sequences are known as clustered regularly interspaced short palindromic repeats (CRISPR) and the enzyme is called CRISPR-Associated (Cas).

In December 2018, Chinese scientist He Jiankui announced that he had used CRISPR-Cas9 technology to edit the genes of embryonic twin girls, an intervention he maintains will help protect the twins from human immunodeficiency virus infection. 

The announcement sparked an international debate about the ethics and safety of such research.

WHO is establishing the expert advisory group to examine the scientific, ethical, social and legal challenges associated with human gene editing. The group will assess the state of human gene editing research and its applications, the technology’s potential uses, and societal attitudes to the technology. 

Based on this work, the group will advise the WHO Director-General on potential oversight mechanisms for research into, and application of, human gene editing technology, and will make recommendations in that regard.

https://www.who.int/ethics/topics/gene-editing/call-for-members/en/

## Tobacco packaging breakthrough

Thailand has become the first country in Asia to adopt plain packaging for tobacco products. 

Legislation requiring plain packaging for tobacco products will enter into force on 10 September 2019. 

Under the new rules, cigarettes will be sold in drab brown coloured packs, free of any logos or images except for graphic health warnings, which are already obligatory in Thailand, as are textual warnings. Thailand also bans tobacco advertisement and promotion, restricts sponsorship and forbids the sale of tobacco products to people less than 20 years of age as well as the sale of single cigarettes. 

Despite these control efforts the prevalence of tobacco use remains high in Thailand where, according to its 2017 national *Smoking and drinking behaviour survey*, roughly one in every five adults in the country smokes. The same survey reported that 37.7% of males aged 15 and above smoked, compared with 1.7% of females in the same age group. 

Plain packaging of tobacco products is recommended under the Guidelines for the Implementation of Articles 11 and 13 of the WHO Framework Convention on Tobacco Control (WHO FCTC), a legal treaty that aims to protect present and future generations against the devastating health and socio-economic impacts of tobacco use. The introduction of plain packaging is expected to further boost the country’s tobacco control efforts.

“Thailand’s bold steps against tobacco – the single most important cause of preventable deaths worldwide – is commendable and reflects the country’s earnest efforts in promoting health and well-being of its people,” said Dr Poonam Khetrapal Singh, Regional Director of the WHO South-East Asia, congratulating Thailand on its new tobacco legislation.

http://www.searo.who.int/mediacentre/releases/2018/1704/en/

## WHO Director-General pushes for end of polio

WHO Director-General Tedros Adhanom Ghebreyesus underlined WHO’s commitment to the final push to eradicate polio on a 4-day visit to Afghanistan and Pakistan, the only two countries where wild poliovirus cases were reported last year.

Together with WHO Regional-Director for the Eastern Mediterranean Dr Ahmed Al-Mandhari, Dr Tedros met with heads of state and senior government officials in both countries and witnessed first-hand WHO-supported health programmes.

Dr Tedros also visited the Emergency Operations Centre for Polio Eradication in Islamabad, Pakistan, where he commended the work of government and partners as “one team under one roof” and highlighted the critical importance of Pakistan working closely with Afghanistan to prevent cross-border transmission.

“We must all give our best on this last mile to eradicate polio once and for all. My wish for 2019 is for zero polio transmission. You have WHO’s full support to help reach every child and stop this virus for good,” Dr Tedros said.

As recently as 30 years ago, wild poliovirus paralysed more than 350 000 children in more than 125 countries every year. In 2018 there were 29 reported cases in just two countries – Afghanistan and Pakistan.

Polio eradication requires high immunization coverage everywhere to block transmission. Unfortunately, some children are still missing out on polio vaccination for several reasons, including lack of infrastructure, remote locations, population movement, conflict and insecurity and resistance to vaccination.

Failure to eradicate polio from these last remaining strongholds could result in a resurgence of the disease, with as many as 200 000 new cases predicted worldwide every year within 10 years.

## India to tackle air pollution

India has launched an action plan to cut air pollution in the country’s 102 worst affected cities. Announced on 10 January, the *National clean air plan* targets cuts in industrial and vehicular emissions as well as a reduction in the burning of biomass. The overall aim of the plan is to achieve a 20-30% reduction in particulate matter concentrations by 2024, relative to levels reported in 2017.

According to the WHO global ambient air quality database, 14 of the 15 cities with the worst air pollution in the world are in India. A study published in Lancet Planetary Health in December 2018, reported that toxic air claimed 1.24 million lives in India in 2017.

**Cover photo**

Village women carry dried cow dung cakes in the Teliarganj area on the outskirts of the city of Allahabad, in the state of Uttar Pradesh, India. Cow dung cakes are a major source of domestic fuel for rural households and a source of indoor air pollution.

**Figure:**
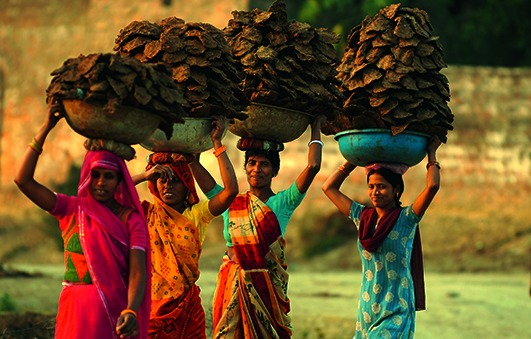
AFP/Getty Images Diptendu Dutta.

## Road traffic injuries leading killer of young

Road traffic injuries are now the leading killer of children and young people aged 5-29 years, according to *Global status report on road safety 2018*. 

The WHO report, which was released in December last year, found that road traffic crash-related deaths continue to rise, with 1.35 million fatalities in 2016, the latest year for which data are available. Pedestrians, cyclists and motorcyclists are the main victims and those living in developing countries are worst affected.

“These deaths are an unacceptable price to pay for mobility,” said Dr Tedros at the launch of the report.

He noted that there are proven measures to reduce road traffic injury, including strategies to reduce speeding and address drinking and driving, among other dangerous behaviours. Also, safer infrastructure such as dedicated lanes for cyclists and motorcyclists can also help, as can improved vehicle standards such as those that mandate electronic stability control. Finally, prompt, effective post-crash care is vital to improving survival. 

## The Prince Mahidol Award Conference 

This annual conference, held in Bangkok, Thailand, focuses this year on noncommunicable diseases (NCDs), under the banner: “The Political Economy of NCDs: A Whole-of-Society Approach”. The overall aim of the conference is to propose solutions at national and global levels to accelerate implementation of NCD prevention and control measures. 

Policy-makers and other senior government officials attend this annual conference, as well as representatives from civil society organizations, international organizations and development partners, universities and industries.

http://pmac2019.com/site/conferenceprogram

**Looking ahead**

4 February – World Cancer Day 2014

6–8 February – WHO Symposium on the Future Digital Health Systems in the European Region, Copenhagen, Denmark

13 – 14 February – First Food and Agriculture Organization, World Health Organization and African Union International Conference on Food Safety, Addis Ababa, Ethiopia.

3 – March – International Ear and Hearing Care Day

